# Construction of a nurse shark (*Ginglymostoma cirratum*) bacterial artificial chromosome (BAC) library and a preliminary genome survey

**DOI:** 10.1186/1471-2164-7-106

**Published:** 2006-05-03

**Authors:** Meizhong Luo, HyeRan Kim, Dave Kudrna, Nicholas B Sisneros, So-Jeong Lee, Christopher Mueller, Kristi Collura, Andrea Zuccolo, E Bryan Buckingham, Suzanne M Grim, Kazuyo Yanagiya, Hidetoshi Inoko, Takashi Shiina, Martin F Flajnik, Rod A Wing, Yuko Ohta

**Affiliations:** 1 Arizona Genomics Institute, Department of Plant Sciences, University of Arizona, Tucson, AZ 85721, USA; 2 College of Life Sciences and Technology, Huazhong Agricultural University, Wuhan 430070, China; 3 University of Maryland, Department of Microbiology and Immunology, 655 West Baltimore Street, BRB3-052, Baltimore, MD 21201, USA; 4 Department of Molecular Life Science, Division of Basic Medical Science and Molecular Medicine, Tokai University School of Medicine, 143 Shimokasuya, Isehara, Kanagawa 259-1143, Japan

## Abstract

**Background:**

Sharks are members of the taxonomic class Chondrichthyes, the oldest living jawed vertebrates. Genomic studies of this group, in comparison to representative species in other vertebrate taxa, will allow us to theorize about the fundamental genetic, developmental, and functional characteristics in the common ancestor of all jawed vertebrates.

**Aims:**

In order to obtain mapping and sequencing data for comparative genomics, we constructed a bacterial artificial chromosome (BAC) library for the nurse shark, *Ginglymostoma cirratum*.

**Results:**

The BAC library consists of 313,344 clones with an average insert size of 144 kb, covering ~4.5 × 10^10 ^bp and thus providing an 11-fold coverage of the haploid genome. BAC end sequence analyses revealed, in addition to LINEs and SINEs commonly found in other animal and plant genomes, two new groups of nurse shark-specific repetitive elements, NSRE1 and NSRE2 that seem to be major components of the nurse shark genome. Screening the library with single-copy or multi-copy gene probes showed 6–28 primary positive clones per probe of which 50–90% were true positives, demonstrating that the BAC library is representative of the different regions of the nurse shark genome. Furthermore, some BAC clones contained multiple genes, making physical mapping feasible.

**Conclusion:**

We have constructed a deep-coverage, high-quality, large insert, and publicly available BAC library for a cartilaginous fish. It will be very useful to the scientific community interested in shark genomic structure, comparative genomics, and functional studies. We found two new groups of repetitive elements specific to the nurse shark genome, which may contribute to the architecture and evolution of the nurse shark genome.

## Background

Bacterial artificial chromosome (BAC) libraries are indispensable for many applications in genomic studies [1-3]. BAC-end sequences have been used to develop sequence-tagged connector (STC) frameworks [4,5], to survey genome structures [6], and for comparative analysis of gene structure and synteny. Although the whole-genome shotgun method has been used to produce genome draft sequences [7], mapped BACs usually have been required to provide a framework for sequence assembly and templates to complete the sequences of complex genomes [3,5,8]. BACs and BAC-based maps have also been used in sequencing of targeted genome regions [9], chromosomal landing or positional cloning [10], genome function investigation [11], and evolutionary and comparative studies [12]. Through BAC sequencing, a full-length avian androgen receptor gene was identified, which had not been detected with conventional methods [13].

Sharks belong to the phylogenetic taxon comprising the oldest jawed vertebrates, the cartilaginous fish, which diverged from the common ancestor of all other jawed vertebrates 460–520 million years ago [14]. Genomic and genetic studies of this group, in comparison to representative species in other vertebrate taxa, will allow us to theorize about the fundamental genetic, developmental, and functional characteristics in the common ancestor of all jawed vertebrates. This ancient taxon is of particular interest to us since it is the oldest group of living animals having an adaptive immune system with underlying molecules and mechanisms similar to those of mammals [15]. While most sharks and other cartilaginous fish have large genome sizes (~80% of species studied have larger genome sizes than human, with some up to 5 times as large), the nurse shark genome size is relatively small at 4 × 10^9 ^bp/haploid genome, only slightly larger than that of humans (3.4 × 10^9^). Thus, the nurse shark is a candidate to become a model species in the biomedical and genomics fields. However, most of the shark genes and intergenic regions are much larger than those of mammals, and thus large-insert genomic libraries are essential to obtain sufficient genomic information. Previously, BAC libraries of other cartilaginous fish (clearnose skate and horn shark)were described by Miyake and Amemiya [1]. In this paper we report the construction and characterization of a publicly-available nurse shark BAC library and carry out a preliminary genome survey.

## Results and discussion

### BAC library construction

We constructed a nurse shark BAC library, consisting of a total of 313,344 clones that were deposited in 816 barcode-ordered 384-well microtiter plates. To complete this large library, approximately 20 ligations were performed. Since colonies with a diameter of <1.5 mm did not grow well in the freezing media, only those colonies with a diameter of >1.5 mm on selection-agar plates were picked.

### Insert size distribution

**Figure 1:**
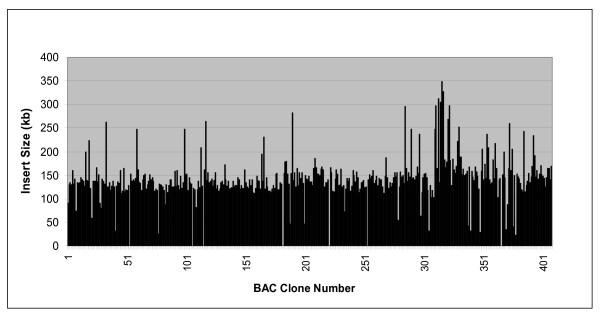
Insert size distribution of the 408 sampling BAC clones. The 408 sampling BAC clones were selected by picking one clone from A01 position of every other 384-well plate of the library and arranged in the same order of the library plates. Vertical bars indicate the sampling clones with inserts. The clones that failed to yield DNA or do not contain inserts are represented by blanks.

To evaluate the quality of the BAC library, we first analyzed the insert sizes of 408 sampling BAC clones with *Not *I (cutting GCGGCCGC sequence), which liberates the inserts from the BAC vector (Figure 1). The 408 sampling BAC clones were selected by picking one clone from A01 position of every other 384-well plate of the library and arranged in the same order as the library plates. Of the 408 BAC clones, 6 did not yield detectable DNA. Of the remaining 402 clones, only one had no insert, indicating a negligible empty-vector rate (1/402). While most clones produced only one *Not *I insert band, ~16% of the clones produced more than one insert band due to the presence of internal *Not *I sites. Eight such clones produced a single or very few small insert bands with high DNA densities disproportional to the DNA density of the vector band, suggesting co-migration of multiple repetitive bands. In fact, when these clones were digested with *Swa *I (cutting ATTTAAAT sequence) that also liberates the inserts, they generated single large-insert bands (e.g. 194 kb). These clones may be of interest because they might contain large repetitive and structurally distinctive regions with extremely high GC content. The average insert size of the 402 clones is 144 kb, and thus the entire BAC library covers ~4.5 × 10^10 ^bp (313,344 × 144,000 bp). The nurse shark genome content was reported to be ~8 pg DNA per cell [16], ~4 × 10^9 ^bp per haploid genome. Thus, our BAC library covers ~11 haploid genome equivalents. Approximately 94% of clones contain inserts greater than 110 kb. Although multiple ligations were used, no significant difference was observed between different library segments corresponding to the different ligations except that one segment of 14,976 clones, corresponding to the library plates 628 to 666 (number 314 to 333 in Figure 1), has very large inserts. This segment resulted from two parallel ligations and has an average insert size of 209 kb.

### BAC end sequence analysis

We sequenced the first 96 of the above 408 sampling clones at both ends. One hundred seventy-seven BAC end sequences (BES) were obtained with an average high-quality base-pair (bp) number of 548 bp ([GenBank:CZ549372~CZ579549], excluding [GenBank:CZ549507] which is the vector sequence), covering a total of ~100,000 bp. The average GC content of the BES is 41%, ranging from 26–66%. Sequences from other parts of the nurse shark genome (~300 kb) have an average of 42–45% GC content (YO, MFF; personal observation), which is consistent with this finding. It is worth noting that the GC content was calculated in windows of the BES lengths, and some smaller regions may contain higher GC content. Indeed, we previously detected simple repeats with a high GC content (70–80%) in a region of the Major Histocompatibility Complex (MHC) class I gene [17]. Analysis of the BES also revealed 4 sequences containing significantly long microsatellite repeats (simple sequence repeats, SSRs): [GenBank:CZ549546] (AG)_9_, [GenBank:CZ549467] (AG)_19_, [GenBank:CZ549532] (AT)_9 _and [GenBank:CZ549547] (AC)_14_. However, no significant tri-nucleotide or longer motifs of SSRs were found in our BES. The CpG dinucleotide frequency was under-represented (the observed value is only 1/4 of the expected one) while the AA and TT dinucleotide frequencies were over-represented in this set of BES.

BlastN and BlastX searches of these BES against the 'nr' database in GenBank and tBlastX and tBlastN searches against an in-house collection of conserved domains of non-LTR retroelements were carried out with an E-value of below 1e-10. The Blast analysis results are classified in Table 1 (BlastN results can be found in our web site [18]). Nine BES share a significant similarity with various shark genomic sequences; one [GenBank:CZ549423] is similar to 18S ribosomal RNA; two showed hits to zebrafish and *Tetraodon *genomic sequences that remain 'unclassified'; and one hundred and two showed 'No Hits'. Twenty-nine and five BES contained LINEs and SINEs respectively, indicating that these repetitive elements are major components of the nurse shark genome. Most of them hit regions of published nurse shark genomic sequences, especially to an intron of the *LMP7-like *pseudogene, and non-coding sequences from genes in other shark species (*RAG *[19] and *HOX *[20]), and the 3'-untranslated region (3'UTR) of a non-classical class I gene, *UAA-NC1 *[17] that contains a retrotransposon. Most of the LINEs contain partial open reading frames (ORFs) encoding reverse transcriptases related to the CR1 family, first found in chicken [21]. The high representation of CR1-like SINEs/LINEs is thus likely to be a common feature of shark genomes. In the sandbar shark (*Carcharhinus plumbeus*), a total of 7 CR1-like SINE/LINE elements were found in the 9.4 kb intergenic region of *RAG1 *and *RAG2 *[22].

### NSRE1 and NSRE2 analysis

**Figure 2:**
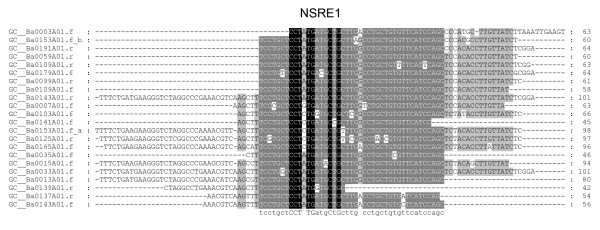
NSRE1 analyses. NSRE1 core nucleotide sequences were aligned using ClustalW. GC_Ba0153A01.f sequence contains two NSRE1 elements (marked as a and b). Shaded color correlates the degree of nucleotide conservation (e.g. black; identical in all sequence). Consensus sequences are shown under the alignment in lower cases.

**Figure 3:**
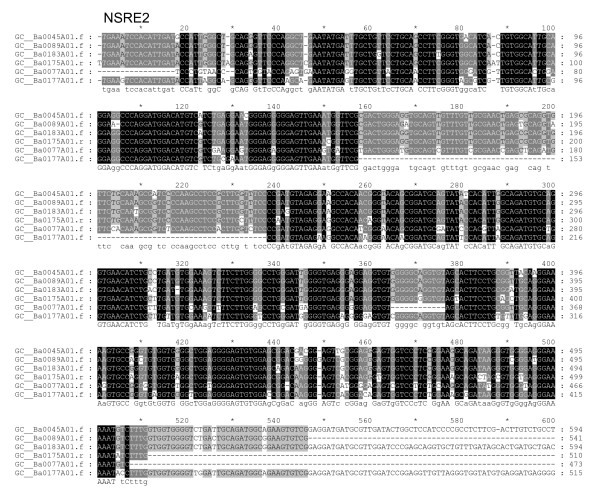
NSRE2 analysis. NSRE2 full-length BES sequences were aligned using ClustalW. Shaded color correlates the degree of nucleotide conservation (e.g. black; identical in all sequence). Consensus sequences are shown under the alignment in lower cases.

**Figure 4:**
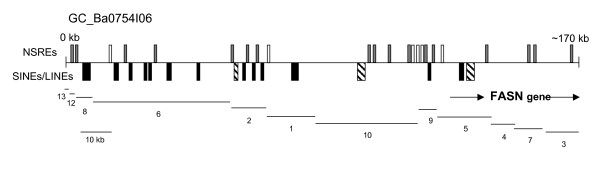
NSRE1 (top, gray box) and NSRE2 (top, open box) were mapped on the draft sequence of the BAC clone GC_Ba0754I06 along with SINEs/LINEs (bottom, hatched box) and CR1-like SINEs/LINEs (bottom, black box). Transcriptional orientation of a partial fatty acid synthase (FASN) gene, including at least 27 exons, is marked with arrows from 5' to 3' direction. Contigs and their numbers are shown under the map. A 10 kb scale is shown on the left bottom.

The remaining 29 BES and one other [GenBank:CZ549472] that also contains a LINE element are of interest. They hit only nurse shark BAC draft sequences when searched against 'htgs' (high-throughput genomic sequence) database in GenBank. When BlastN searches were performed using the whole BES set as the database and the BES as queries, they were categorized into two groups designated as NSRE1 (nurse shark repetitive element 1) and NSRE2 (24 and 6 for NSRE1 and NSRE2 respectively, Figures 2 and 3). Searching against the 'nr' database in GenBank revealed that 21 of 24 NSRE1 sequences hit the nurse shark *UAA-NC1 *cDNA ([GenBank:AF357922]) in the 5'UTR (nt positions 2–103), and 22 of 24 NSRE1 sequences hit the nurse shark LMP7 pseudogene, exon 4 ([GenBank:AF357928]) at nt positions 3274–3350 (see Additional file 1). The 698 bp-sequence of GC_Ba0153A01.f [Genbank:CZ549510] hit the subject regions in two query regions (nt positions 1–60 and 538–635), showing two NSRE1 in one BES. Two BAC clones (GC__Ba0143A01 and GC__Ba0109A01) hit the subject regions at both ends. When the subject sequence [GenBank: AF357922] nt positions 2–103 region was used as a query to search the 'htgs' database in GenBank, it only hit all the 9 nurse shark BAC draft sequences then present in the GenBank in multiple regions with an average of 7.2 hits/BAC, from a minimum of 3 to a maximum of 11 (data of Oct. 2, 2005). Searching the NSRE2 sequence [GenBank:CZ549534] against the 'htgs' database in GenBank found an average of 3.4 hits/BAC on 8 of the 9 nurse shark BAC draft sequences ranging from a minimum of 1 to a maximum of 5. These results indicate that NSRE1 and NSRE2 are repetitive elements, and NSRE1 is more frequent in the nurse shark genome than NSRE2. BlastX searches of both NSRE1 and NSRE2 did not detect any protein matches. To further sample the representation and organization of NSRE1 and NSRE2 elements in the nurse shark genome including coding and non-coding regions, we sequenced the BAC clone GC_Ba0754I06. The draft sequence of this clone revealed seventeen NSRE1 and six NSRE2 in a ~170 kb region dispersed among SINEs/LINEs repetitive elements. All identified repetitive elements are found most abundantly in regions outside of the gene (e.g. fatty acid synthase FASN, Figure 4).

**Figure 5:**
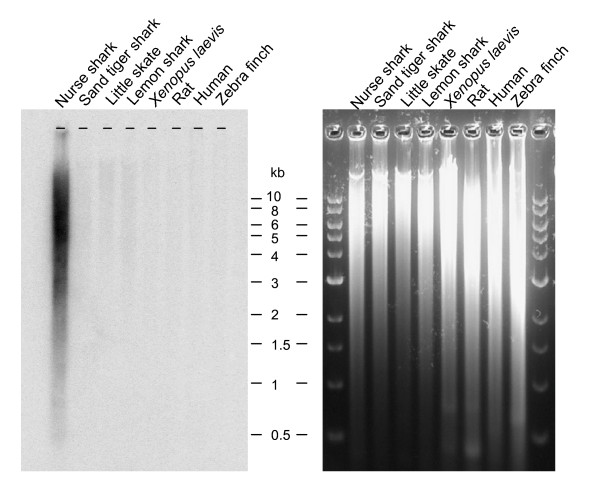
Southern blotting with the NSRE1 'overgo' probe. Genomic DNAs from various species (denoted at the top of the blot) were digested with *Hin *dIII and separated on an agarose gel. The 1 kb size standards were loaded on both sides of the gel. The ethidium bromide-stained image is shown as a loading control on the right of the blot.

To confirm that these repetitive sequences were present in a high copy number in the nurse shark genome and to test for their existence in related animals, we did Southern blotting and library screening using 'overgo' probes of NSRE1 and NSRE2. Both the NSRE1 and NSRE2 probes hybridized to the nurse shark genomic DNA, but not to DNA from other elasmobranchs (sand tiger shark, little skate, and lemon shark), *Xenopus laevis*, rat, human and zebra finch (Figure 5 for NSRE1, data not shown for NSRE2). Both the NSRE1 and NSRE2 hybridization signals are smear, indicating that these elements are highly repetitive and dispersed in the nurse shark genome, consistent with our sequence analysis results above. From a library screening of 36,864 clones, many colonies hybridized with different intensities (data not shown), perhaps correlating with the copy numbers within the BAC clones. Our data suggest that the expansion of NSRE1 and NSRE2 repetitive elements occurred after the divergence of nurse shark from other shark lineages. However, we examined only distantly related shark species in this study. Closely related shark species, belonging to the same family (Orectolobiformes) as nurse shark (e.g. wobbegong shark, bamboo shark), must be examined for the presence/absence of these repetitive elements. It is possible that different, unique repetitive elements will be found in other shark species.

### BAC library screening with gene-specific probes

To further assess the quality and demonstrate the utility of the BAC library, we screened the entire BAC library with gene-specific probes. These probes are listed in Table 2. With all single-copy gene probes except *TAP1 *(transporter associated with antigen processing) and *Ring3*, we obtained 6–28 positive clones (Table 2, library screening). A similar number of positive clones was observed when we used the *Factor B *probe, present in two tandem copies in the nurse shark genome. We further confirmed the positive clones by colony hybridization (Table 2, colony hybridization). Most of the gene probes except *CD83 *[23] resulted in 8–19 true positives, consistent with the 11x coverage calculated from the average insert size and total number of clones. The low percentage (50%) of true positives from MHC class I and *CD83 *may be due to weakly hybridizing false-positives, which appeared only after long film exposures. In some cases, we observed multiple weaker signals in the vicinity of the stronger signals in the same double-spotting pattern, presumably due to carryover during filter production. In fact, the low percentage (62%) of true positives for the *TAP1 *probe was most probably due to such carryover of positive clones into neighboring wells. Once we carefully selected putative positive clones for *TCR *probes (i.e. only selected the strongest signals among the neighboring signals), the percentage of true positives increased (see Table 2, 89–91% *TCR*s). *TAP1 *and *Ring3 *are members of large gene families containing conserved domains, which might cross-hybridize to other family members and thus result in higher number of positive clones than expected. With the *LMP7 *probe, 19 positive clones seem to be low for a multi-copy gene, however we have found at least 2 pseudogenes containing only small fragments of the gene [17]. These results showed that most genetic regions tested in this study (except *CD83*) were well represented in the BAC library.

Unlike most other species, the nurse shark MHC genes are much (3–5 times) larger than those of mammals and have even larger distances in intergenic regions [17]. Previously, we constructed a genomic cosmid library with an average insert size of ~40 kb. However, most cosmid clones contain at most a single gene (data not shown). In this study, we used several linked genes *(TAP1*, MHC class I, class II, *Factor B, LMP7, LMP2 *[17,24], and *Ring3 *(unpublished data)) to quickly glean the number of genes in a single BAC clone; we found up to four genes in a single BAC clone, making physical mapping possible. Thus far, our analysis has convincingly shown that the BAC library is a useful tool (and perhaps the only way) to obtain genetic information for this species.

## Conclusion

We report in this paper a large insert, deep-coverage and high-quality BAC library for a cartilaginous fish that will be very useful to the scientific community for gene isolation, genetic analysis, and comparative genomics. We found two new groups of repetitive elements, designated as NSRE1 and NSRE2, which are specific to the nurse shark genome. These repetitive elements may contribute to the architecture and evolution of the nurse shark genome. The BAC library, HDR filters and individual clones are available to the public from the Arizona Genomics Institute's BAC/EST Resource Center [18].

## Methods

### Isolation of high molecular weight DNA

Blood was obtained from the nurse shark individual "Yellow" using a Heparinized 18G 1 1/2" needle from the caudal vein. To obtain ~30 micrograms of DNA per 80-μl agarose plug, approximately 4 × 10^6 ^erythrocytes were embedded in 1% InCert agarose (FMC, Rockland, ME) prepared in 1/2x PBS and molded in ~200 Plug Molds (BIO-RAD, Hercules, CA). Twenty plugs were then submerged in 50 ml of cell lysis solution (1% lithium dodecyl sulfate, 10 mM Tris, pH8.0, 100 mM EDTA, pH8.0) and incubated overnight at 37°C with occasional swirling. The cell lysis solution was replaced with 50 ml 20% NDS (0.2% N-lauroylsarcosine, 2 mM Tris, pH9.0, 0.14M EDTA, pH9.0), and DNA plugs were shaken gently at room temperature for two hours, and then kept at 4°C [25].

### BAC vector preparation

We used a modified version of the BAC vector pBeloBAC11, pIndigoBAC536Swa. A first modified version of pBeloBAC11 [GenBank:U51113], pIndigoBAC536, was a gift from Dr. H. Shizuya of Caltech. pIndigoBAC536 has the internal *Eco *R1 site of pBeloBAC11 destroyed so that the unique *Eco *R1 site in the multiple cloning sites can be used for cloning, and also contains a random point mutation in the *lac*Z gene that provides colonies with a darker blue-color on X-gal/IPTG selection. We further inserted two *Swa *I sites (ATTTAAAT) near and internal to the two *Not *I sites of pIndigoBAC536 (this new version is named pIndigoBAC536Swa) to facilitate insert-size estimation of clones from GC-rich organisms (Luo et al, unpublished data). We then cloned this single-copy BAC vector pIndigoBAC536Swa into the high-copy vector pGEM-4Z (this composed high-copy plasmid is named pAGIBAC1) to facilitate the preparation of the single-copy BAC vector as we did for pIndigoBAC536 (the composed high-copy pIndigoBAC536-pGEM-4Z plasmid is named pCUGIBAC1) [26]. pCUGIBAC1 is available through Clemson University Genomics Institute [27] and pAGIBAC1 is available through Arizona Genomics Institute [18]. The linearized and dephosphorylated single-copy BAC vector pIndigoBAC536Swa can be prepared from the high-copy pAGIBAC1 according to our previously published method [28].

### Generation and size selection of large DNA fragments for BAC cloning

Large genomic DNA fragments for BAC cloning were prepared according to our previously published method [28]. The DNA-agarose plugs were washed thoroughly with TE buffer (10 mM Tris/1 mM EDTA, pH8.0) and stored in 70% ethanol at -20°C. A desired number of DNA plugs were transferred to TE buffer the day before use and kept at 4°C overnight. The DNA plugs were test-digested with various amount of *Hin *dIII (1–50U) for 20 minutes at 37°C to optimize partial digestion conditions and the fragmented DNAs were separated on 1% agarose gels by Pulsed Field Gel Electrophoresis (PFGE) (CHEF Mapper, BIO-RAD) at 1–50 sec linear ramp, 6 volts/cm, 14°C in 0.5X TBE buffer for 18–20 hours. Bulk digestions were then carried out using the conditions that produced the most DNA fragments in the range of 100–400 kb. Fragmented DNAs were separated on a 1% CHEF gel in the same conditions described above. DNA fractions ranging from 150–250 kb and 250–350 kb were excised from the gel and subjected to a second size selection on a 1% CHEF gel at 4 sec constant time, 6 volts/cm, 14°C in 0.5X TBE buffer for 18–20 hours. DNA fragments were electroeluted with dialysis tubing as described by Strong et al [29] or with an Electro-eluter Model 422 (BIO-RAD) following the manufacture's instructions. DNA concentrations were determined on subsequent agarose gels.

### Ligation and transformation

One hundred to two hundred nanograms of size-selected DNA fragments were ligated with 20 nanograms of dephosphorylated BAC vector in a 100 μl of volume at 16°C overnight. The ligation reactions were terminated at 65°C for 15 min, and the ligation products were desalted in 0.1 M glucose : 1% agarose cones for 1.5 hours on ice as described by Atrazhev and Elliott [30] and electroporated into the *E. coli *strain DH10B T1 phage resistant (F-*mcr*A Δ(*mrr*-*hsd*RMS-*mcr*BC) φ80d*lac*Z ΔM15 Δ*lac*X74 *deo*R *rec*A1 *end*A1 *ara*D139 Δ(*ara, leu*) 7697 *gal*U *gal*K λ-*rps*L *nup*G) electrocompetent cells (Invitrogen, Carlsbad, CA). Transformants were grown on LB plates supplemented with 12.5 mg/L chloramphenicol, 80 mg/L X-gal (5-bromo-4-chloro-3-indolyl-beta-D-galactoside or 5-bromo-4-chloro-3-indolyl-beta-D-galactopyranoside) and 100 mg/L IPTG (Isopropyl-beta-D-thiogalactoside or Isopropyl-beta-D-thiogalactopyranoside) at 37°C overnight.

### Library arraying and high density replica (HDR) filters

A total of 313,344 individual recombinant clones (white color on X-gal plates) were picked robotically (Genetix, New Milton, UK) and arrayed into 816 barcode-ordered 384-well microtiter plates containing freezing media (10 g/L Bacto tryptone, 5 g/L Bacto yeast extract, 10 g/L NaCl, 36 mM K_2_HPO_4_, 13.2 mM KH_2_PO_4_, 1.7 mM Na-citrate, 6.8 mM (NH_4_)_2_SO_4_, 4.4% glycerol, autoclaved and added filter-sterilized MgSO_4 _solution to the final concentration of 0.4 mM) supplemented with 12.5 mg/L of chloramphenicol. After an overnight incubation at 37°C, empty wells were back-filled manually and duplicate copies were replicated. The master library and the two copies were then stored in -80°C freezers at different locations. The whole BAC library was gridded onto 17 22.5 cm × 22.5 cm Hybond N+ membrane filters (Amersham, Piscataway, NJ) in high density, double spots, and 4 × 4 patterns with Genetix Q-bots (Genetix). Each 22.5 cm × 22.5 cm filter supports 18,432 clones in duplicate in 6 fields. The filters were placed on LB media supplemented with 12.5 mg/L of chloramphenicol and incubated overnight at 37°C. The filters were then soaked in 0.5N NaOH/1.5M NaCl for 7 min, in 1.5M NaCl/0.5M Tris-HCl (pH8) for 7 min, air dried for 1–2 hours, soaked in 0.4N NaOH for 20 min, in 20x SSPE for 7 min, and air-dried overnight.

### DNA analysis of BAC clones

BAC DNAs were extracted with Tomtec Quadra 96 model 320 (Tomtec, Hamden, CT) in a 96-well format at AGI. The 408 sampling BAC clones were selected by picking one clone from A01 position of every other 384-well plate of the library and arranged in the same order of the library plates. Inserts were liberated by digesting with *Not *I or *Swa *I and their sizes were determined on CHEF gels.

### BAC end sequencing

BAC DNAs were sequenced at both ends using BigDye Terminator v.3 (Applied Biosystems, ABI, Foster City, CA) according to manufacturer's instruction. The T7 primer (5' TAA TAC GAC TCA CTA TAG GG 3') was used as the "forward" primer and the BES_HR primer (5' CAC TCA TTA GGC ACC CCA 3') was used as the "reverse" primer. Cycle sequencing was performed using PTC-200 thermal cyclers (MJ Research, Waltham, MA) in a 384-well format with the following regime: 150 cycles of 10 sec at 95°C, 5 sec at 55°C, and 2.5 min at 60°C. After the cycle-sequencing step, the DNA was purified by magnetic beads, CleanSeq (Agencourt, Beverly, MA) according to manufacturer's instruction. Samples were eluted into 20 μl of water and separated on ABI 3730xl DNA capillary sequencers with default conditions. Sequence data was collected by data collection software (Applied Biosystems), extracted using sequence analysis software (Applied Biosystems) and transferred to a UNIX workstation. Sequences were base-called using the program Phred [31,32]; vector and low-quality (Phred value <16) sequences were removed by CROSS_MATCH [31,32].

### Bioinformatics analyses of sequences

Similarity searches against public GenBank and in-house database were carried out using the Blast algorithm. Composition analyses as well as searches for inverted repeats were done using the programs "composition" and "palindrome" respectively, both of which are included in the package EMBOSS [33]. SSR were searched using the software "Sputnik" [34].

### NSRE alignment

The nucleotide sequences of both NSRE1 and NSRE2 were aligned using ClustalW. NSRE1 motifs were extracted from BES and full-length BES sequences were aligned for NSRE2.

### Southern blotting for NSREs

Five μg genomic DNAs were digested with 80 units of restriction enzyme *Hin *dIII for 6 hours at 37°C. DNA fragments were separated in a 0.8% agarose gel by electrophoresis and blotted onto a nylon membrane. Overlapping oligonucleotide (overgo) hybridization was performed according to Ross et al [35] with modifications. The NSRE1 and NSRE2 overgo probes were designed from the sequence [GenBank:AF357922] nt positions 2–103 region and sequence [GenBank:AF357928] nt position 4406–4529 region respectively. Primers used for NSRE1 are: 5' TCT CGG CCC GAA ACG TCA GCT TTC 3' and 5' AGC ATC AGA GGA GCA CGA AAG CTG 3'. Primers used for NSRE2 are: 5' TGC TGT TCC TGC AAC CTT CGG GTA 3' and 5' AAT GCC ACA ACG ACG CTA CCC GAA 3'. Each set of primers overlaps by 8 base pairs. Probes were labeled with both ^32^P-dCTP and ^32^P-dATP using Klenow enzyme. Hybridization was carried out overnight in a solution containing 1% Bovine Serum Albumin (BSA), 1 mM EDTA pH8.0, 7% SDS, 0.5M sodium phosphate at 60°C. Membranes were washed in 4x SSC, 0.1% SDS at room temperature, followed by 1.5x SSC, 0.1% SDS at 60°C. Membranes were exposed to screens and scanned using the phosphor imager.

### BAC sequencing and assembly

The BAC clone GC_Ba0754I06 that covered 170 kb was bidirectionally shotgun sequenced with an average redundancy of about 6, which was sufficient for assembly and analysis of the entire sequence using previously established procedures [36]. The draft sequence was searched using Blast2 for NSRE1 and NSRE2, BlastN and BlastX against the 'nr' database in GenBank for SINEs/LINEs and CR1-like SINEs/LINEs, respectively. During the BlastX search, at least 27 exons were identified with significant similarity to other species' fatty acid synthase (FASN) exons (E-values of 3e-56 and 3e-46 to chicken [GenBank:AAB46389] and rat [GenBank:AAA41145], respectively).

### BAC library screening

The seventeen HDR filters of the BAC library were pre-hybridized in high-stringency hybridization solution (50% Formamide, 6x SSC, 0.5% SDS, 5x Denhardt's solution) [37] supplemented with 100μg/ml of denatured salmon sperm DNA for ~4 hours at 42°C. Probes (50 ng) were radiolabeled using the random-priming method (Roche, Indianapolis, IN) or by incorporating α^32^P-dCTP in the polymerase chain reaction (PCR) [38]. Hybridization was performed overnight at 42°C and membranes were washed at room temperature in pre-warmed (42°C) 2x SSC/1% SDS for 20 minutes, followed by washing in 0.2x SSC/0.1% SDS for 20 minutes at 65°C. Membranes were exposed to X-ray film for various lengths of time to obtain positive signals and the desired background. General protocols for high-density BAC library filter screening and address determination of positive signals are publicly available from our website [18]. Putative positive clones were re-spotted on nylon membranes for colony hybridization to confirm true positives. For hybridization with NSRE overgo probes, we performed in the same hybridization solution as described above for Southern Blotting. Filters were washed in 4x SSC, 0.1% SDS at room temperature, followed by 0.75x SSC, 0.1% SDS at 60°C, exposed to screens, and scanned using the phosphor imager.

## Authors' contributions

RAW, MFF, ML and YO participated in the design of this study. ML constructed the BAC library. ML, YO, HK, DK, NBS, S-JL, CM, KC, AZ, EBB and SMG participated in library characterization and data analysis. KY, HI, and TS participated in sequencing and contig assembly of the BAC clone GC_Ba0754I06. ML, MFF, and YO prepared this manuscript. All authors have read and approved the final manuscript.

## Supplementary Material

Additional File 1 Analysis of NSRE1 and NSRE2 sequences. GenBank accession numbers containing NSRE1 and NSRE2 identified in this study are listed in this table. Some of them also matched the two nurse shark sequences [GenBank:AF357922] and [GenBank:AF357928]; the length and the % identities at the nucleotide level are also shown. Click here for file

## Figures and Tables

**Table 1:** The classification of BES.

Classification	Number of BES
LINE	29
SINE	5
NSRE1	24
NSRE2	6
Shark genomic	9
18S Ribosomal	1
Unclassified	2
No Hits	102
Total	177*

* One BES contains both LINE and NSRE1.

**Table 2:** Number of gene-specific clones in the nurse shark BAC library Single copy genes.

Single copy genes				
	Number of positive clones		%	

Genes	Library Screening	Colony hybridization	Colony hyb./Library scr.	References

TAP1	60	37	62%	[17]
Ring3	72	62	86%	Unpublished
MHC class I	20	10	50%	[17]
CD83	6	3	50%	[23]
Gici-11*	28	19	68%	[37]
LMP2	18	NA	NA	[17]
TCRβ	9	8	89%	Unpublished
TCRδ	11	10	91%	Unpublished

Possibly two genes in tandem or close vicinity				

Genes	Library screening	Colony hybridization	Colony hyb./Library scr.	References

MHC class IIα	14	12	86%	[39]
MHC class IIβ	13	9	69%	[40]
Factor B	16	14	87%	[24]

Multiple genes				

Genes	Library screening	Colony hybridization	Colony hyb./Library scr.	References

LMP7**	41	19	46%	[41]

NA: not available

*several genes, but our 3'UTR specific probe only identifies one gene

**only one complete and functional gene (i.e. many pseudogenes)
