# Supplementary material for: Construction of a nurse shark (Ginglymostoma cirratum) bacterial artificial chromosome (BAC) library and a preliminary genome survey
Source: BMC Genomics. 2006 May 3;7:106. doi: 10.1186/1471-2164-7-106 (PMC1513397; doi:10.1186/1471-2164-7-106)
Supplement: Additional File 1 — Analysis of NSRE1 and NSRE2 sequences. GenBank accession numbers containing NSRE1 and NSRE2 identified in this study are listed in this table. Some of them also matched the two nurse shark sequences [GenBank:AF357922] and [GenBank:AF357928]; the length and the % identities at the nucleotide level are also shown. [file 1471-2164-7-106-S1.doc]

Supplemental table: Analysis of NSRE1 and NSRE2 sequences

| Group | Accession Number | Sequence Name* | Match on AF357922 | | | Match on AF357928 | | |
| --- | --- | --- | --- | --- | --- | --- | --- | --- |
|  |  |  | Match Region | Match Length (bp) | % Identities / Gaps | Match Region | Match Length (bp) | % Identities / Gaps |
| NSRE1 | CZ549418 | GC__Ba0051A01.r | no match | 0 |  | 3288-3332 | 45 | 93/0 |
|  | CZ549374 | GC__Ba0003A01.f | 28-82 | 54 | 87/0 | 3290-3349 | 60 | 91/0 |
|  | CZ549378 | GC__Ba0007A01.f | 34-93 | 63 | 90/0 | 3274-3342 | 69 | 91/0 |
|  | CZ549384 | GC__Ba0013A01.f | 3-82 | 80 | 83/0 | 3288-3349 | 62 | 93/0 |
|  | CZ549403 | GC__Ba0033A01.f | 3-103 | 101 | 88/0 | 3276-3346 | 71 | 94/0 |
|  | CZ549405 | GC__Ba0035A01.f | 37-82 | 46 | 86/0 | 3289-3349 | 61 | 95/0 |
|  | CZ549426 | GC__Ba0059A01.r | 40-99 | 60 | 90/0 | 3276-3332 | 57 | 94/0 |
|  | CZ549464 | GC__Ba0099A01.r | 40-100 | 61 | 90/0 | 3276-3332 | 57 | 94/0 |
|  | CZ549467 | GC__Ba0103A01.f | 35-100 | 66 | 86/0 | 3276-3337 | 62 | 90/0 |
|  | CZ549471 | GC__Ba0109A01.f | 40-97 | 58 | 89/0 | 3276-3332 | 57 | 94/0 |
|  | CZ549472** | GC__Ba0109A01.r | 40-102 | 63 | 90/0 | 3276-3332 | 57 | 91/0 |
|  | CZ549484 | GC__Ba0121A01.r | no match | 0 |  | no match | 0 |  |
|  | CZ549488 | GC__Ba0125A01.r | 35-100 | 66 | 83/0 | 3276-3349 | 74 | 87/1 |
|  | CZ549500 | GC__Ba0137A01.r | 28-81 | 54 | 85/0 | 3291-3350 | 60 | 90/0 |
|  | CZ549502 | GC__Ba0139A01.r | 18-59 | 42 | 88/0 | 3294-3346 | 53 | 92/0 |
|  | CZ549503 | GC__Ba0141A01.f | 35-79 | 45 | 88/0 | 3276-3349 | 74 | 89/1 |
|  | CZ549505 | GC__Ba0143A01.f | 27-82 | 56 | 85/0 | 3290-3349 | 60 | 91/0 |
|  | CZ549506 | GC__Ba0143A01.r | 3-103 | 101 | 88/0 | 3276-3349 | 74 | 95/0 |
|  | CZ549510*** | GC__Ba0153A01.f | 2-100  40-99 | 99  60 | 87/1  86/0 | 3276-3337;  3276-3332 | 62  57 | 93/0  89/0 |
|  | CZ549522 | GC__Ba0165A01.f | 4-100 | 97 | 86/1 | 3280-3333 | 54 | 92/0 |
|  | CZ549549 | GC__Ba0191A01.r | 40-103 | 64 | 90/0 | 3276-3332 | 57 | 92/0 |
|  | CZ549536 | GC__Ba0179A01.f | 40-103 | 64 | 85/0 | 3276-3324 | 49 | 93/0 |
|  | CZ549477 | GC__Ba0115A01.f | no match | 0 |  | no match | 0 |  |
|  | CZ549386 | GC__Ba0015A01.f | 39-97 | 59 | 84/0 | 3276-3333 | 58 | 93/0 |
| NSRE2 | CZ549414 | GC__Ba0045A01.f | no match | 0 |  | no match | 0 |  |
|  | CZ549441 | GC__Ba0077A01.f | no match | 0 |  | no match | 0 |  |
|  | CZ549453 | GC__Ba0089A01.f | no match | 0 |  | no match | 0 |  |
|  | CZ549533 | GC__Ba0175A01.r | no match | 0 |  | no match | 0 |  |
|  | CZ549540 | GC__Ba0183A01.f | no match | 0 |  | no match | 0 |  |
|  | CZ549534 | GC__Ba0177A01.f | no match | 0 |  | 4406-4529 | 124 | 88/0 |

*GC_Ba is the name of the shark BAC library; four digits designate the number of the 384-well plates; A01 presents the clone location on each 384-well plate; f and r indicate the forward and reverse sequences respectively. **Also contains a LINE element. ***Two query regions hit the subject regions.
